# Bright Light Decreases Peripheral Skin Temperature in Healthy Men: A Forced Desynchrony Study Under Dim and Bright Light (II)

**DOI:** 10.1177/07487304221096948

**Published:** 2022-06-20

**Authors:** R. Lok, T. Woelders, M. J. van Koningsveld, K. Oberman, S. G. Fuhler, D. G. M. Beersma, R. A. Hut

**Affiliations:** * Chronobiology Unit, Groningen Institute for Evolutionary Life Sciences, University of Groningen, Groningen, the Netherland; † Department of Psychiatry and Behavioral Sciences, Stanford University, Palo Alto, California, USA; ‡ University of Groningen, Leeuwarden, the Netherlands

**Keywords:** light, sleep-wake-related variation, circadian, forced desynchrony, thermoregulation, proximal skin temperature, distal skin temperature

## Abstract

Human thermoregulation is strictly regulated by the preoptic area of the hypothalamus, which is directly influenced by the suprachiasmatic nucleus (SCN). The main input pathway of the SCN is light. Here, thermoregulatory effects of light were assessed in humans in a forced desynchrony (FD) design. The FD experiment was performed in dim light (DL, 6 lux) and bright white light (BL, 1300 lux) in 8 men in a semi-randomized within-subject design. A 4 × 18 h FD protocol (5 h sleep, 13 h wake) was applied, with continuous core body temperature (CBT) and skin temperature measurements at the forehead, clavicles, navel, palms, foot soles and toes. Skin temperature parameters indicated sleep-wake modulations as well as internal clock variations. All distal skin temperature parameters increased during sleep, when CBT decreased. Light significantly affected temperature levels during the wake phase, with decreased temperature measured at the forehead and toes and increased navel and clavicular skin temperatures. These effects persisted when the lights were turned off for sleep. Circadian amplitude of CBT and all skin temperature parameters decreased significantly during BL exposure. Circadian proximal skin temperatures cycled in phase with CBT, while distal skin temperatures cycled in anti-phase, confirming the idea that distal skin regions reflect heat dissipation and proximal regions approximate CBT. In general, we find that increased light intensity exposure may have decreased heat loss in humans, especially at times when the circadian system promotes sleep.

Thermoregulation is critical for mammalian survival. While low body temperature can lead to diminished enzymatic reactions and cellular functions, dangerous elevations in body temperature can cause protein denaturation (Matsumi et al., 1994; Withman, 1988). The human body contains a heat producing core and heat dissipating shell (Aschoff, 1955; Aschoff and Wever, 1958; Rushmer et al., 1966). At rest, body heat production depends on metabolic activity of inner organs, such as liver, brain, heart, and contraction of skeletal muscles (Aschoff and Wever, 1958; Burse, 1979; Eichna et al., 1951). Heat dissipation occurs through the skin (Rushmer et al., 1966). Round shaped parts of the skin, such as fingertips and toes, are especially efficient in heat loss (Aschoff, 1971; Refinetti and Menaker, 1992). Heat (re)distribution from the core to extremities occurs through blood transport (Johnson and Proppe, 1996). In addition to capillaries, distal skin regions contain arteriovenous anastomoses (AVAs) and glomerular structures of tortuous thin vessels (retia venosa; Lossius et al., 1993). AVAs are thick-walled vessels between arterioles and venules, allowing significant circulation and heat transfer to the environment (Vanggaard et al., 2012), while the large surface area of the retia venosa also facilitates heat loss (Grahn et al., 2011).

Temperature measured in the heat producing core (core body temperature [CBT]) is maintained within an inter-threshold range, with a set-point near 37 °C. The anterior hypothalamus and preoptic area receive temperature information from the core through hypothalamic receptors and from the environment through skin receptors (Boulant, 2000; Morf and Schibler, 2013). Both processes influence the thermoregulatory set-point. Under normal physiological conditions, temperature can only increase or decrease by a few tenths of a degree Celsius without reaching the threshold that triggers autonomic thermoregulatory responses (Pitoni et al., 2011). However, the heat producing set-point itself fluctuates over the course of a day due to varying activity levels of the suprachiasmatic nucleus (SCN). The SCN is the most important internal pacemaker and its activity levels fluctuate in a circadian manner. Direct projections from the SCN to the preoptic area of the hypothalamus exist (Buijs et al., 1993; Vrang et al., 1995), resulting in daily fluctuations in CBT levels. Highest temperatures occur in the evening, while the nadir is reached in the early morning (Aschoff and Wever, 1981; Aschoff et al., 1967; Morf and Schibler, 2013).

The SCN is entrained by light signals from the retinohypothalamic tract and, in turn, synchronizes circadian body temperature cycles to environmental light-dark cycles. Direct effects of light exposure on core and skin temperature regulation, independent of its phase shifting properties, suggest that light effects on thermoregulation might not necessarily be SCN dependent (te Kulve et al., 2016). On the other hand, light exposure in the evening, when CBT is high, elevates CBT levels (Dijk et al., 1991), while morning bright light (BL) exposure, when CBT is low, also increases CBT (Aizawa et al., 1997). This suggests that light exposure might reduce SCN-induced thermoregulatory fluctuations.

To determine whether light effects on thermoregulation are SCN dependent, a forced desynchrony (FD) design can be used. The principle of a FD design is that sleep-wake periods are scheduled with a duration that deviates sufficiently from 24 h such that it falls outside the range of circadian entrainment by light. This allows the internal pacemaker to free run (i.e., following its intrinsic circadian period) throughout scheduled sleep and wakefulness (Kleitman and Kleitman, 1953), resulting in multiple combinations of homeostatic sleep drive levels and circadian clock time. Under the assumption of free running of the circadian pacemaker with a period close to 24 h, it is possible to mathematically disentangle sleep-wake related and circadian clock time effects on parameters of interest (Dijk et al., 1992; Hiddinga et al., 1997). In this experiment, an FD design conducted in DL and BL was used to study whether light effects on human thermoregulation depend on the circadian clock time or sleep-wake-related variation.

## Materials and Methods

### Subjects

For an elaborate version of the “Materials and Methods” section, including participant information and proof-of-principle of the BL FD conditions (in which cortisol data indicate no evidence for non-uniform phase progression in the BL FD), please see Lok et al. (2022). In short, healthy subject s (n=8) were included to participate in a within-subject FD design, conducted once under polychromatic white dim light (DL; 6 lux, 5 melanopic lux) and once in BL (1300 lux, 1050 melanopic lux) conditions. All participants were non-sleep-deprived males between the ages of 20 and 30 years (average ± standard error of the mean (SEM); 24.0 ± 1.16). All participants provided written informed consent and received financial compensation for participation. The study protocol, screening questionnaires, and consent forms were approved by the medical ethics committee of the University Medical Center Groningen (NL54128.042) and were in agreement with the Declaration of Helsinki (2013).

### Power Calculations

To assess if presented results were acquired with sufficient validity, a power calculation was conducted. Cajochen et al. (2005) reported a significant effect of BL exposure on distal proximal gradient (DPG) with an effect size of 5.16. With alpha set to 0.05 and power to 0.8, a total of 3 participants per light condition should be included to find a statistically significant difference in DPG. Cajochen et al. (2005) tested effects of light on DPG in the evening hours, and therefore included bright light induced melatonin suppression, which in turn can induce temperature changes. Therefore, an additional power calculation was conducted using (Aizawa and Tokura, 1998). They reported a significant effect of BL exposure on tympanic temperature, with an effect size of 4.16. With alpha set to 0.05 and power to 0.8, 3 participants per condition are necessary to reach statistical significance.

### Protocol

**Figure 1.**
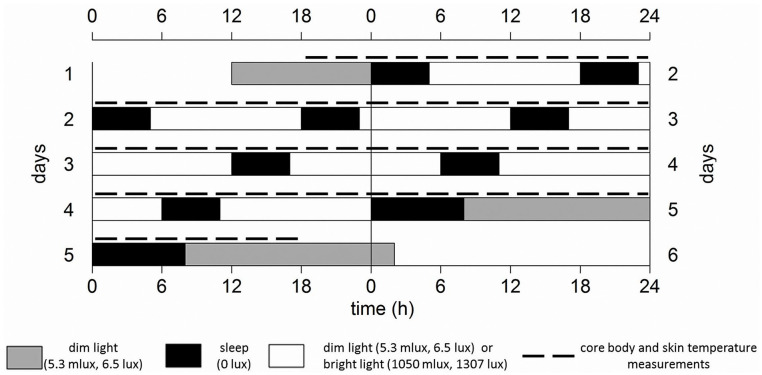
Schematic representation of the experiment design, for an individual with an HSon at 0000 h, double-plotted. Gray bars indicate dim light (6 lux) conditions preceding/following the forced desynchrony protocol. Black bars represent intervals for sleep (5 h, except for the last sleep attempt which is allowed to last for 8 h) with lights off (0 lux), while white bars represent wakefulness in either polychromatic white dim (6 lux, 5 melanopic lux) or bright light (1300 lux, 1050 melanopic lux) conditions. Dotted lines indicate core body and skin temperature measurements. The protocol lasted for 72 h, therefore comprising a full beat (3 × 24 h = 72 h, 4 × 18 h = 72 h).

Participants arrived at the human isolation facility of the University of Groningen 10 h before habitual sleep onset (HSon; assessed with the Münich Chronotype Questionnaire [MCTQ]; Roenneberg et al., 2003). Upon arrival, participants were equipped with DS1922L Ibuttons (Thermochron, Australia, temperature range of –40 °C to +85 °C, 0.0625 °C resolution, with ±0.5 °C accuracy) for measuring skin temperature on the forehead (*T*_forehead_), navel (*T*_navel_), right and left subclavicular regions (*T*_subclavicular_), both hand palms (*T*_palms_), at the arch underneath the feet (*T*_sole_), and in the left and right pulp of the first toe on the side of the big toe (*T*_toes_). Subjects ingested an e-Celcius performance logger (Bodycap, France) to measure CBT (30-sec interval). Participants would receive a new pill every 18 h, or earlier if the pill had left the body before 18 h had passed. The FD protocol commenced with 5 h for sleep (in darkness, with sheets), starting at HSon. Participants were woken up under polychromatic white light of either DL or BL and remained awake under these light conditions for the next 13 h (for detailed α-opic lux values, see Suppl. Fig. S3 and Table S1, FD alertness, this issue). Thereafter, participants were instructed to go to bed and light intensities were set to 0 lux. The 18-h FD cycle, consisting of 5 h for sleep and 13 h for wakefulness, was repeated 4 times, resulting in a 72-h FD protocol (4 times 18 h exactly matches 3 times 24 h). Room temperature was kept constant at 21 °C. During scheduled wake intervals, physical exercise was not allowed, resulting in minimal activity. Participants were free in the choice of clothing, but no differences between light conditions were observed. After completion of the first FD protocol, subjects were allowed to go home and returned after at least 3 weeks to participate in the same FD protocol under opposing light conditions. The order of light conditions was counterbalanced. The experiment was conducted between February and May 2018 (Figure 1).

### Data Preprocessing: CBT

CBT data were collected for all subjects under both light conditions, allowing for a within-subject comparison. Outliers (absolute consecutive temperature change exceeding 2 °C) were omitted.

### Data Preprocessing: Skin Temperature

Due to a technical malfunction, the complete skin temperature dataset was only collected during the second visit of all subjects. Skin temperature parameters were therefore compared between individuals (4 during DL and 4 others during BL), resulting in a between-subject comparison. Outliers (absolute consecutive temperature change exceeding 2 °C, 195 data points [2%] in total) were omitted. Distal (*T*_distal_) and proximal (*T*_proximal_) skin temperature were calculated as the average temperature of *T*_palms_ and *T*_toes_, and of *T*_subclavicular_, respectively. The DPG (*T*_DPG_) was calculated as *T*_distal_ minus *T*_proximal_ (Kräuchi et al., 1997a).

### Statistics

RStudio (version 1.4.1717, running on R version 4.1.0) was used for statistics and graphs. Sleep-wake-related contributions to body temperature measures were quantified by grouping and averaging data in 1-h bins according to time since lights on/wake-up. To determine circadian variation, solely original data collected during light exposure were calculated as a function of circadian phase (in degrees relative to Dim Light Melatonin Onset (DLMO)) in 30-degree bins. For optimal clarity, corresponding time of day (h) is depicted for both wake-duration-related and circadian variation. This has been depicted for an individual with wake-up time of 0800 and a DLMO of 1900. Linear mixed models were constructed with light condition as independent variable, time since sleep offset and circadian phase as a fixed effect (categorical variable) and added interaction terms between time since sleep offset and light, and circadian phase and light condition. Although room temperature was kept constant, there were some differences between light conditions (DL; 21.05 ± 0.18, BL; 21.23 ± 0.20 [mean ± SD], Suppl. Fig. S1 and Table S1). To statistically control for this, human isolation facility room number was also included as a random effect in all linear mixed models. Critical 2-sided significance level alpha was 0.05 for all statistical tests. To ensure sufficient sample size (*n* ≥ 4) for each combination of ‘time since sleep offset’ and ‘circadian clock phase’, we constructed a separate linear mixed model to calculate significance of the interaction terms of these variables with light condition. Contrast analyses (comprising of a Tukey post hoc test corrected for multiple testing [Tukey correction], package “lsmeans”) were conducted on all combinations of circadian time and time since sleep offset. Contrasts were constructed in 90-degree bins, with wake-dependent changes in bins of 2.6 h during light exposure and 2.5 h during dark exposure. Significant contrasts are depicted in 3-dimensional graphs, in which circadian variation is represented on the *x*-axis (in 90-degree bins), wake-duration-related variation is presented on the *y*-axis, and BL scores subtracted from DL scores are presented on the z-axis (with colors indicating the direction and magnitude of statistically significant light effects). Combinations of wake-duration-related variation and circadian variation that contain data of fewer than 4 individuals are considered underpowered and presented as “missing data” in gray. Effects of time since lights on/off were tested together (effects of sleep pressure) as well as separately (effects of sleep and sleep pressure effects during wake, respectively). To estimate light effects during the time course over the projected day, sleep was predicted to start 2 h after DLMO (Burgess et al., 2003; coinciding with circadian phase 30) and last for 8 h (until circadian phase 160), after which 13 h of wakefulness commenced. All values are displayed as mean +/- SEM, unless noted otherwise.

## Results

### Original Data

**Figure 2.**
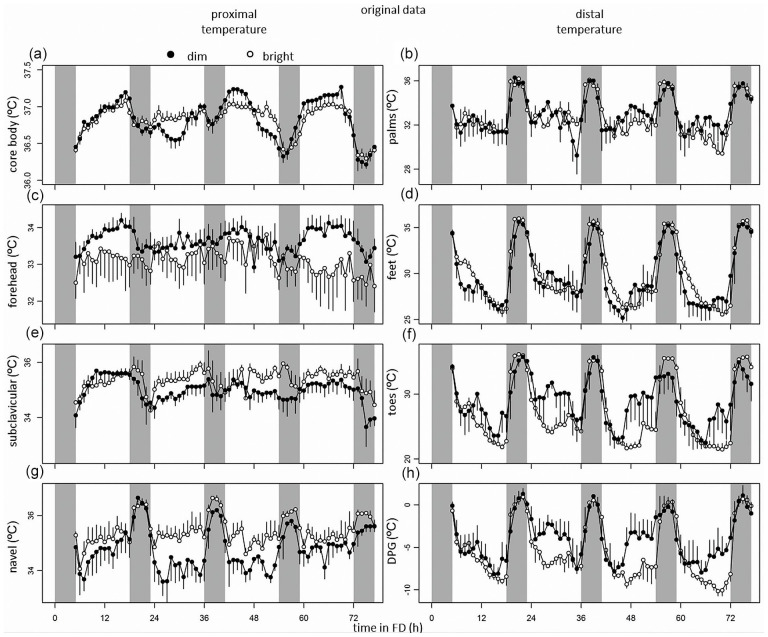
From top to bottom, data of core body temperature, and skin temperatures measured at the palms, forehead, feet, subclavicular region, toes, navel, and DPG. Time course of CBT (a), skin temperature measured at the palms (b), forehead (c), feet (d), clavicles (e), toes (f), navel (g), and DPG (h) during the forced desynchrony protocol. Data represent mean ± standard error of the mean, with 4 subjects per group for skin temperature measurements, and 8 subjects per group for CBT measurements. Black dots indicate data collected in dim light, and white dots represent data collected in bright light. Abbreviation: DPG = distal-proximal gradient.

Original data plotted on a continuous time axes indicate clear effects of both sleep-wake-related variation and circadian modulations (Figure 2). Correlations among CBT and skin temperature correlates can be found in Figure S2.

### Sleep-Wake- and Light-Dark-Cycle-Related Variation (Process S)

**Figure 3.**
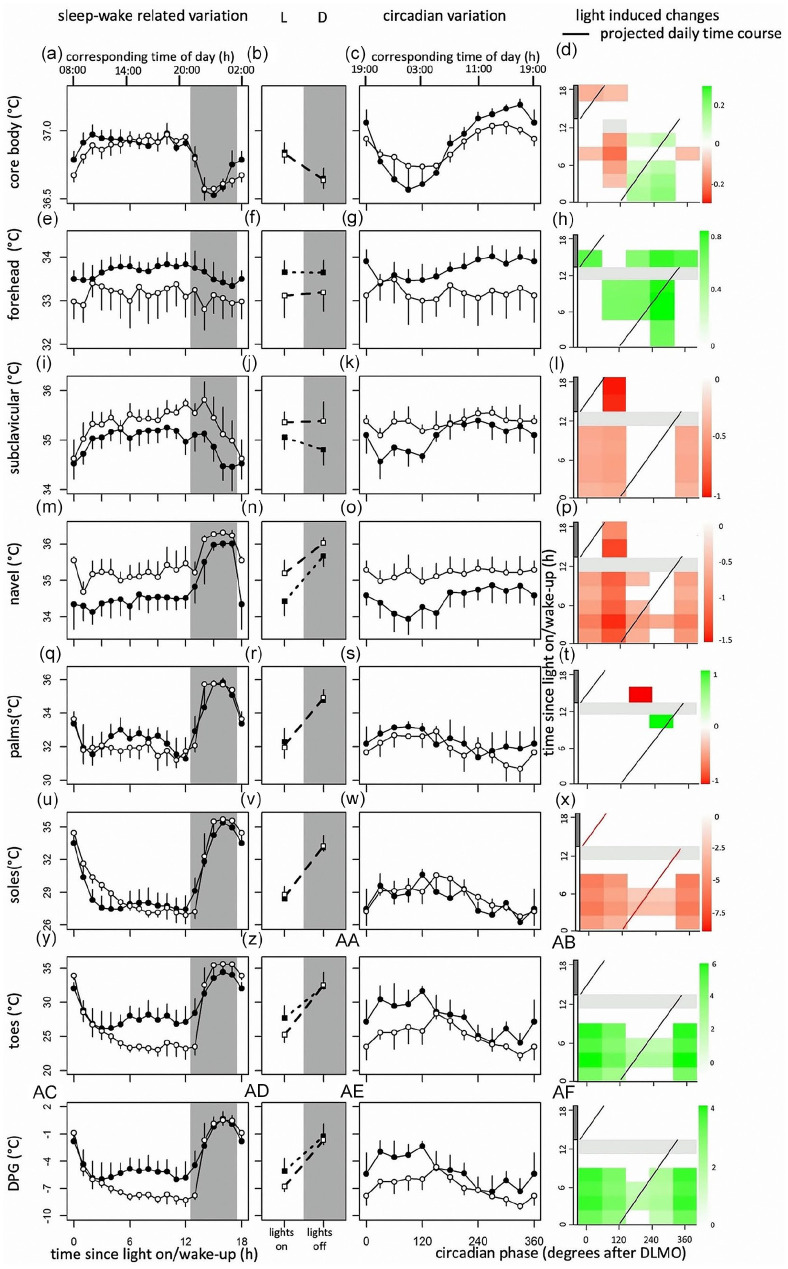
From top to bottom, data of core body temperature, and skin temperatures measured at the forehead, subclavicular region, navel, palms, feet, toes, and distal-proximal gradient. Original data from Figure 2 replotted as time since lights on or off (a, e, i, m, q, u, y, ac) and circadian phase in degrees after Dim Light Melatonin Onset (c, g, k, o, s, w, aa, ae), for CBT, *T*_forehead_, *T*_subclavicular_, *T*_navel_, *T*_palms_, *T*_sole_, *T*_toes_, and *T*_DPG_, respectively. Corresponding time of day (h) is depicted on the top axis. Contrast analysis describing light-induced increase for all combinations of circadian clock phase and time since lights on for CBT (d), *T*_forehead_ (h), *T*_subclavicular_ (l), *T*_navel_ (p), *T*_palms_ (t), *T*_sole_ (x), *T*_toes_ (ab), and *T*_DPG_ (af). Red squares indicate light-induced increases in temperature, while green rectangles represent light-induced decreases. Data represent mean ± standard error of the mean, with 4 subjects per group for skin temperature measurements, and 8 subjects per group for CBT measurements. 13 h after lights on, lights were turned off (0 lux), and subjects were instructed to go to sleep. Black dots indicate data collected in dim light, white dots represent data collected in bright light, and black and white squares represent averages over all data points under DL and BL, respectively. Red line indicates the projected time course over a regular day. Shaded areas represent scheduled sleep (at 0 lux). Significant differences between light conditions (*p* < 0.05) are indicated by colored rectangles (d, h, l, p, t, x, ab, af). Gray rectangles indicate combinations of wake-duration-related variation and circadian clock phase containing data of less than 4 individuals (d, h, l, p, t, x, ab, af). Abbreviations: CBT = core body temperature; DL = dim light; BL = bright light; DPG = distal-proximal gradient. Color version of the figure is available online.

There were significant modulations over the wake-sleep cycle in all thermoregulatory parameters (CBT, *T*_subclavicular_, *T*_navel_, *T*_palms_, *T*_sole_, *T*_toes_, *T*_DPG_), except for *T*_forehead_ (Figure 3, Tables 1 and 2). When comparing all intervals of enforced waking with intervals of sleep, there was a significant effect of sleep on CBT, *T*_subclavicular_, *T*_navel_, *T*_palms_, *T*_toes_, and *T*_DPG_, with CBT rhythms indicating decreased amplitude during the dark compared to the light phase. *T*_navel_, *T*_palms_, *T*_sole_, *T*_toes_, and *T*_DPG_ levels all depicting an increased amplitude during the dark phase, while *T*_subclavicular_ levels decreased, and *T*_forehead_ levels were comparable between dark and light phases (Figure 3, Tables 1 and 2). Light exposure significantly affected temperature measured at *T*_forehead_, *T*_subclavicular_, *T*_navel_, *T*_toes_, and *T*_DPG_, with *T*_forehead_, *T*_toes_ and *T*_DPG_ showing a decreased amplitude during BL relative to DL, while *T*_subclavicular_ and *T*_navel_ showed increased temperature levels (Figure 3, Tables 1 and 2).

### Circadian Control (Process C)

There were significant changes in CBT, *T*_subclavicular_, *T*_navel_, *T*_sole_, *T*_toes_, and *T*_DPG_ depending on internal clock time, with lowest values in CBT, *T*_subclavicular_, and *T*_navel_ and highest values of *T*_sole_, *T*_toes_, and *T*_DPG_ around circadian phase 90 (6 h after DLMO; Figure 3, Tables 1 and 2). *T*_subclavicular_ and *T*_navel_ showed circadian modulation in-phase relative to CBT (particularly in DL), while *T*_palms_, *T*_sole_, *T*_toes_, and *T*_DPG_ all cycled in anti-phase with CBT (Figure 3, Table 1). *T*_forehead_ did not vary with circadian clock phase.

**Table 1.** Summary of statistics of wake-related variation (process S), circadian variation (process C), interactions between process S and light, the interaction between Process C and light, as well as the additive effects of bright light exposure.

	Wake-Duration-Related Variation (Process S)		Circadian variation (Process C)		Interaction (Process S × Light)		Interaction (Process C × Light)		Additive Effect of Bright Light	
Core body temperature	*F*_17,544_,*p*	**44.67, <0.0001**	*F*_11,550_,*p*	**137.09, <0.00001**	*F*_17,544_,*p*	1.58, >0.05	*F*_11,550_,*p*	***11.83, <0.0001***	*F*_1,544_,*p*	0.54, >0.05
*T*_forehead_	*F*_17,544_,*p*	0.91, >0.05	*F*_11,550_,*p*	1.34, >0.05	*F*_17,544_,*p*	*0.38*, >*0.05*	*F*_11,550_,*p*	*0.51, >0.05*	*F*_1,544_,*p*	**53.31, <0.00001**
*T*_subclavicular_	*F*_17,544_,*p*	**8.67, <0.00001**	*F*_11,550_,*p*	**6.51, <0.001**	*F*_17,544_,*p*	*1.33*, >*0.05*	*F*_11,550_,*p*	***1.95, <0.05***	*F*_1,544_,*p_,_*	**128.61, <0.00001**
*T*_navel_	*F*_17,544_,*p*	**8.66, <0.0001**	*F*_11,550_,*p*	**3.55, <0.0001**	*F*_17,544_,*p*	*0.48*, >*0.05*	*F*_11,550_,*p*	*1.78, >0.05*	*F*_1,544_,*p*	**25.85, <0.0001**
*T*_palms_	*F*_17,544_,*p*	**25.12, <0.0001**	*F*_11,550_,*p*	**2.97, <0.0001**	*F*_17,544_,*p*	*1.22*, >*0.05*	*F*_11,550_,*p*	*0.79, >0.05*	*F*_1,544_,*p*	1.78, >0.05
*T*_sole_	*F*_17,544_,*p*	**22.61, <0.0001**	*F*_11,550_,*p*	**2.67, <0.00001**	*F*_17,544_,*p*	*1.38*, >*0.05*	*F*_11,550_,*p*	*0.96, >0.05*	*F*_1,544_,*p*	1.61, >0.05
*T*_toes_	*F*_17,544_,*p*	**41.97, <0.0001**	*F*_11,550_,*p*	**6.42, <0.0001**	*F*_17,544_,*p*	***3.83***, < ***0.0001***	*F*_11,550_,*p*	***1.93, <0.05***	*F*_1,544_,*p*	**76.01, <0.0001**
*T*_DPG_	*F*_17,544_,*p*	**60.38, <0.0001**	*F*_11,550_,*p*	**10.72, <0.0001**	*F*_17,544_,*p*	***3.30, <0.0001***	*F*_11,550_,*p*	***2.56 <0.001***	*F*_1,544_,*p*	**183.80, <0.00001**

Values from linear mixed models on core body temperature, *T*_forehead_, *T*_subclavicular_, *T*_navel_, *T*_palms_, *T*_sole_, *T*_toes_, and *T*_DPG_.

**Table 2.** Summary of effects of sleep-wake modulations and circadian clock phase on skin and core body temperature.

	Effects of Sleep Compared With Wake	Temperature Levels in BL Compared With DL During Wake	Temperature Levels in BL Compared With DL During Sleep	Internal Clock Amplitude in BL Compared With DL	Internal Clock Phase Relative to CBT Phase	Light-Induced Changes During Projected Daily Time Course
CBT	–	–	0	–		–
*T*_forehead_	0	–	–	0		–
*T*_subclavicular_	0	0	+	0	In-phase	0
*T*_navel_	+	0	+	0	In-phase	+
*T*_palms_	+	0	0	0	Anti-phase	0
*T*_sole_	+	0	0	0	Anti-phase	0
*T*_toes_	+	–	–	0	Anti-phase	–
*T*_DPG_	+	–	–	0	Anti-phase	–

Columns indicate (1) effects of sleep, (2) effects of the light intervention, (3) after effects of the light intervention, (4) internal clock amplitude changes due to the light intervention, (5) phase of internal clock time of skin temperature parameters relative to CBT, and (6) occurrence of light effects during the projected daily time course “–” indicates a decrease, “+” indicates an increase, and “0” indicates unchanged values.

Abbreviations: BL = bright light; DL = dim light; CBT = core body temperature.

### Light-Induced Changes During the Projected Time Course Over a Regular Day

Significant interactions were found between circadian phase, sleep-wake-related variation, and light-induced change in temperature parameters. Visual assessment suggested that light significantly decreases CBT, *T*_forehead_, *T*_toes_, and *T*_DPG_ during the projected daily time course, while *T*_navel_ significantly increases. *T*_subclavicular_, *T*_soles_, and *T*_palms_ are modestly modulated by light exposure during the daily time course. All light effects predominantly occur during 13 h of light exposure and are less pronounced during the subsequent dark phase (Figure 3).

## Discussion

Light effects on human thermoregulation have been assessed previously (Lok et al., 2019), but it remains to be elucidated how these light effects depend on circadian clock phase and sleep-wake-related variation. Here, we show that BL exposure alters levels of CBT at multiple circadian clock phases. High intensity lighting increased temperature levels in proximal regions (*T*_subclavicular_ and *T*_navel_), while *T*_forehead,_ *T*_toes_, and *T*_DPG_ showed decreased temperature levels during BL exposure. These effects occurred both depending on circadian clock phase and sleep-wake-related variation. BL exposure dampened the circadian amplitude in CBT. Proximal skin temperature cycled in phase with CBT, while distal skin temperature parameters fluctuated in anti-phase. Visual assessment suggested that during the projected daily time course, BL decreases CBT, *T*_forehead_, *T*_toes,_ and *T*_DPG_, while *T*_navel_ significantly increases. Other parameters were modestly modulated by light exposure during the projected daily time course.

### Circadian Variation in CBT Levels

CBT is regulated by the circadian pacemaker, which has been assessed in this and other FD protocols (Dijk et al., 1992; Hiddinga et al., 1997; Monk and Carrier, 1998; M. P. Johnson et al., 1992; Wyatt et al., 1999). Fluctuations in CBT levels coincide with rhythms of melatonin production (Cajochen et al., 1998, 2005; Kräuchi et al., 1997b); peak melatonin values are associated with the nadir in CBT, while the subsequent decline in melatonin is followed by an increase in CBT (Cagnacci et al., 1997). The CBT decreasing effect of melatonin is partly caused by increased heat loss due to peripheral vasodilatation by melatonin (Lok et al., 2019). Correlations between melatonin concentrations and CBT levels are confirmed by our results, which show elevated CBT levels when light-induced melatonin suppression occurs. Although higher CBT values were detected immediately after DLMO, significant effects of BL exposure on CBT could also be detected at circadian phases when melatonin is virtually absent. Correlation analyses further indicated additional effects of BL exposure on CBT, independent of melatonin suppression (Suppl. Figs. S3 and S4, and Table S2).

### Correlations Between CBT and Skin Temperature

The current view of the human thermoregulatory system is that *T*_proximal_ (usually measured subclavicularly) reflects CBT, while *T*_distal_ (measured at hands and/or feet) depicts temperature fluctuations in heat dissipating regions (Kräuchi and Wirz-Justice, 1994). With respect to time awake, CBT and *T*_subclavicular_ increase, while *T*_distal_ decreases. This reflects a mode of heat conservation during wakefulness. Overall, this heat-conservation mode seems stronger under BL conditions, possibly due to melatonin suppression. BL decreases temperature measured in *T*_toes_ and *T*_DPG_ and increases *T*_navel_ and *T*_subclavicular_, especially at circadian phases when melatonin can be suppressed by BL. However, CBT, with respect to time awake, remains relatively unaffected by light, which potentially indicates redistribution of body heat content. *T*_forehead_ (unexpectedly) decreases under BL conditions, especially at circadian phases when melatonin is not present under DL condition. Due to its location, *T*_forehead_ could be thought to reflect *T*_proximal_, but *T*_forehead_ displays no circadian variation. Moreover, light effects depending on sleep-wake variation indicate a decrease in temperature, opposed to other *T*_proximal_ parameters, but rather resembling *T*_distal_ temperatures. Other studies also indicated that *T*_forehead_ does not correlate well with proxies of CBT (Sessler and Moayeri, 1990). It is possible that *T*_forehead_ is influenced by brain temperature, with relatively constant levels, explaining the absence of circadian influence. With respect to internal clock time, *T*_proximal_ cycles in phase with CBT, while *T*_distal_ parameter fluctuates in anti-phase (Table 2), suggesting that indeed *T*_proximal_ reflects CBT, while *T*_distal_ indicates heat dissipation. These measurements of circadian clock time fluctuations suggest that *T*_proximal_ and *T*_distal_ reflect CBT fluctuations fitting with current knowledge. Overall, BL effects on skin temperatures suggest enhanced heat conservation, which can only be partly explained through melatonin suppression by light.

### Altered Levels of CBT and Skin Temperature During the Dark Phase

It is known that CBT fluctuations coincide with sleep onset (Gilbert et al., 2004; Liao et al., 2013; Murphy and Campbell, 1997; Raymann et al., 2005; Rogers et al., 2007). Multiple studies have suggested that postural changes influence thermoregulatory levels (Cajochen et al., 1997; Kräuchi et al., 1997a; Moul et al., 2002; Tikuisis and Ducharme, 1996). This is most likely the consequence of a decreased muscle tonus caused by lying down, resulting in less heat generation (Tikuisis and Ducharme, 1996). As heat loss is mostly regulated through AVAs, temperatures increase in *T*_distal_ when CBT decreases, while *T*_proximal_ temperatures increase. In this experiment, however, both *T*_distal_ and *T*_proximal_ show clear increases during sleep. Although results might be masked by blankets used during sleep, increased skin temperature levels could also be the consequence of decreased cutaneous blood flow due to orthostatic vasoconstriction in response to decreased blood pressure as a consequence of lying down (Tanabe and Shido, 1994). Increased blood flow increases the cooling effect of venous blood returning to the core, thus leading to decreased CBT (Mittleman and Mekjavic, 1988; Johnson, 1966). Only *T*_forehead_ remains relatively unaffected by posture changes and blankets, which might be due to the fact that forehead temperature is predominantly influenced by arterial blood temperature, and that vasomotor control is minimized for the skin in the head (Day, 1968).

### After-Effects of Light Exposure During the Dark Phase

Although dark conditions were identical between the DL and BL group, temperature patterns did differ. Increased light exposure may decrease heat loss in humans, especially at times when the circadian system promotes sleep. Other studies also report persistent after-effects of BL exposure, lasting for 4 h or longer (Dijk et al., 1991; Rosenthal et al., 1990). It has been suggested that BL might alter the CBT set-point or influences the onset of vasodilatation (Aizawa and Tokura, 1998), therewith inducing long-term changes in CBT and skin temperature. However, previously conducted studies may have induced phase shifts, since light exposure occurred at times of day that the circadian system was sensitive to light exposure (Dijk et al., 1991; Rosenthal et al., 1990). Light effects presented here are independent of circadian phase shifts, suggesting persistent after-effects even without altering circadian clock phase. These effects might be the consequence of heat storage, which is released during subsequent bed rest (Lee and Haymes, 1995).

### Disturbed Circadian Rhythms Might Alter CBT

Blunted temperature rhythms with varying internal clock time might be the consequence of activity at internal clock phases when sleep is promoted. Night shift simulations, consisting of an abrupt shift of the sleep schedule, also reduce CBT amplitude, without changing melatonin production (Cuesta et al., 2017). This indicates that being awake at circadian phases when sleep is promoted induces CBT changes, independent of light exposure or melatonin production. Furthermore, light exposure during the sleep maintenance window may dampen the amplitude in CBT, as SCN firing rates during these time periods can easily be increased. During the wake-maintenance zone, SCN firing rates are already relatively high, limiting light effects at those times of day due to a ceiling effect. Nocturnal rats that are exposed to continuous light exhibit lower CBT amplitudes (Honma and Hiroshige, 2017), while constant darkness decreased CBT in day-active squirrels (Refinetti and Kenagy, 2018), pigeons show decreased CBT amplitudes after transitioning from light-dark to constant light (Berger and Phillips, 2017), and day-active polar bears at the Arctic decrease CBT amplitude with increasing day length (Durner et al., 2015). BL exposure seems to have similar effects on CBT levels in diurnal and nocturnal animals, suggesting a light-retina-SCN-CBT pathway, since increased firing rate induces sleep in nocturnal animals (Borbély, 1976), while the opposite is true for diurnal animals (Badia et al., 1991).

### Changes During the Projected Daily Time Course

Significant interactions were found between sleep-wake-related variation, circadian clock phase, and light-induced change in temperature parameters. BL exposure significantly lowered levels of CBT, *T*_forehead_, *T*_toes_, and *T*_DPG_. These effects occurred at various combinations of circadian clock phase and time since light on, as well as during the projected daily time course (visually assessed). Lower DPG values have been associated with higher alertness (Cajochen et al., 2005), suggesting a link between daytime-light-induced alertness and thermoregulation (Lok et al., 2020).

### Limitations

The relatively low number of participants could complicate statistical interpretation. However, given the conducted power calculations (described in the “Materials and Methods” section), this seems unlikely. In addition, participants were free in choice of clothing. Although no differences in chosen clothing items were observed between the 2 light conditions, this could have influenced results described here. Finally, the Thermochron iButtons have an accuracy of 0.5 °C, which could significantly influence results reported here. However, other studies reporting light effects on thermoregulation employ similar thermoregulatory measurements with similar accuracy. Moreover, light effects described here exceed the possible inaccuracy of the iButtons (1 °C at the maximum). It is therefore likely that there is a light effect on human skin temperature, especially in regions where this effect is bigger than 1 °C (*T*_navel_, *T*_toes_, and *T*_DPG_).

## conclusion

In conclusion, this FD study shows that BL affects human thermoregulation, as inferred from core body, and proximal and distal skin temperatures. These effects depend on sleep-wake-related variation, internal circadian clock time, and can only partly be explained by light-induced melatonin suppression. Some of these light effects occur during the predicted time course of a regular day and could be advantageous for improving daytime alertness.

## Supplemental Material

sj-docx-1-jbr-10.1177_07487304221096948 – Supplemental material for Bright Light Decreases Peripheral Skin Temperature in Healthy Men: A Forced Desynchrony Study Under Dim and Bright Light (II) Click here for additional data file. Supplemental material, sj-docx-1-jbr-10.1177_07487304221096948 for Bright Light Decreases Peripheral Skin Temperature in Healthy Men: A Forced Desynchrony Study Under Dim and Bright Light (II) by R. Lok, T. Woelders, M. J. van Koningsveld, K. Oberman, S. G. Fuhler, D. G. M. Beersma and R. A. Hut in Journal of Biological Rhythms
