# Supplementary material for: Bright Light Decreases Peripheral Skin Temperature in Healthy Men: A Forced Desynchrony Study Under Dim and Bright Light (II)
Source: J Biol Rhythms. 2022 Jun 20;37(4):417–28. doi: 10.1177/07487304221096948 (PMC9326805; doi:10.1177/07487304221096948)
Supplement: sj-docx-1-jbr-10.1177_07487304221096948 – Supplemental material for Bright Light Decreases Peripheral Skin Temperature in Healthy Men: A Forced Desynchrony Study Under Dim and Bright Light (II) [file sj-docx-1-jbr-10.1177_07487304221096948.docx]

**Title:** Bright light decreases peripheral skin temperature in healthy men: a forced desynchrony study under dim and bright light (II)

**Running title:** Bright causes peripheral vasoconstriction.

**Authors:** R. Lok^1,2,3,^*, T. Woelders^1,3^, M.J. van Koningsveld^1^, K. Oberman^1^, S.G. Fuhler^1^, D.G.M. Beersma^1^, R.A. Hut^1^

**Contact Information: ^1^**University of Groningen, Chronobiology unit, Groningen Institute for Evolutionary Life Sciences, PO box 11103, 9700CC, Groningen, the Netherlands.

**^2^**University of Groningen, Campus Fryslân, Wirdumerdijk 34, 8911 CE, Leeuwarden, the Netherlands.

^3^ Should be considered as joint first author.

^*^ To whom all correspondence should be addressed: Renske Lok, University of Groningen, Chronobiology Unit, Present address: Department of Psychiatry and Behavioral Sciences, Stanford University, 401 Quarry Road, Palo Alto, CA, 94305, [rlok@stanford.edu](mailto:rlok@stanford.edu)

**Supplemental information**

**
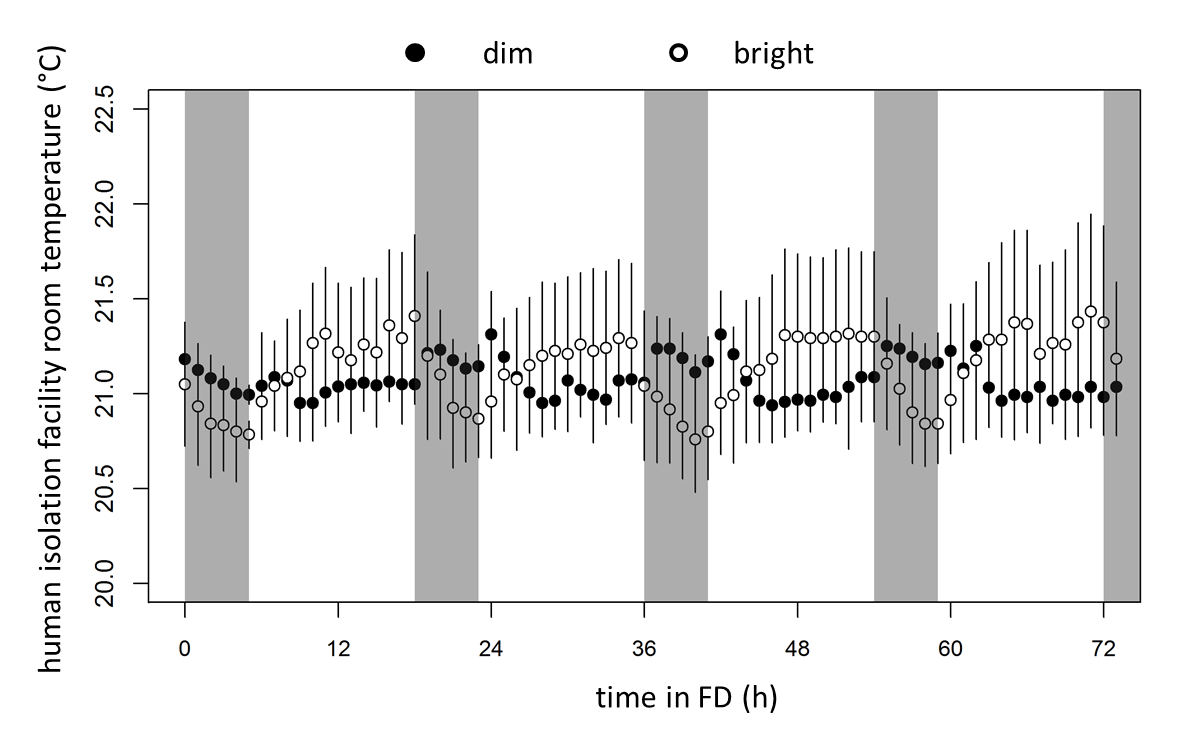
**

**Figure S1:** Room temperature measured during the FD experiment. Data represent mean ± standard error of the mean, with 4 human isolation facility rooms per group. Black dots indicate data collected in dim light, while white dots represent data collected in bright light. There was a significant differences in room temperature (p=0.03, DL; 21.07 ±0.18, BL; 21.12 ± 0.20 (mean ± SD)). To statistically correct for dissimilarities between the temperature in the human isolation facility rooms, room number was included as a random effect in the model.

**Table S1:** Overview of room temperature measured during the FD experiment. Data indicate median ± interquartile range for every individual per light condition.

| Subject | Condition | Temperature (median [IQR]) |
| --- | --- | --- |
| 1 | BL | 21.9 [21.6 – 22.0] |
| 2 | DL | 21.4 [21.3 - 21.4] |
| 3 | DL | 21.0 [20.9 - 21.0] |
| 4 | DL | 20.9 [20.6 - 21.2] |
| 5 | BL | 20.6 [20.4 - 20.7] |
| 6 | BL | 21.4 [21.1 - 21.6] |
| 7 | DL | 21.0 [21.0 - 21.1] |
| 8 | BL | 21.2 [21.1 - 21.3] |

**
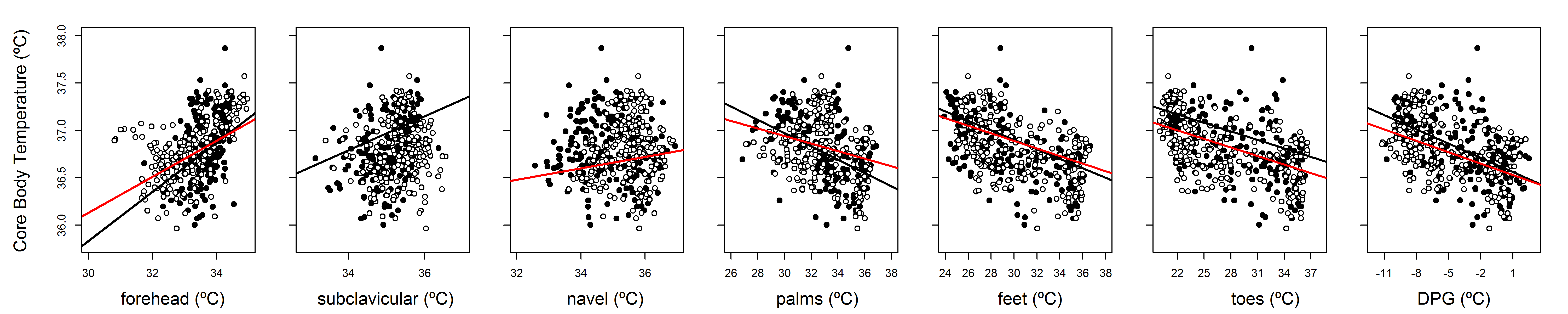
**

**Figure S2: Correlations between skin temperature measured at the forehead, subclavicular region, navel, hand palms, feet, first pulp of the toe, distal-proximal gradient and Core Body Temperature (ºC).** Lines indicate significant correlations, with black and red lines following data collected in dim and bright light respectively. Data is averaged in bins of 0.5 ºC.


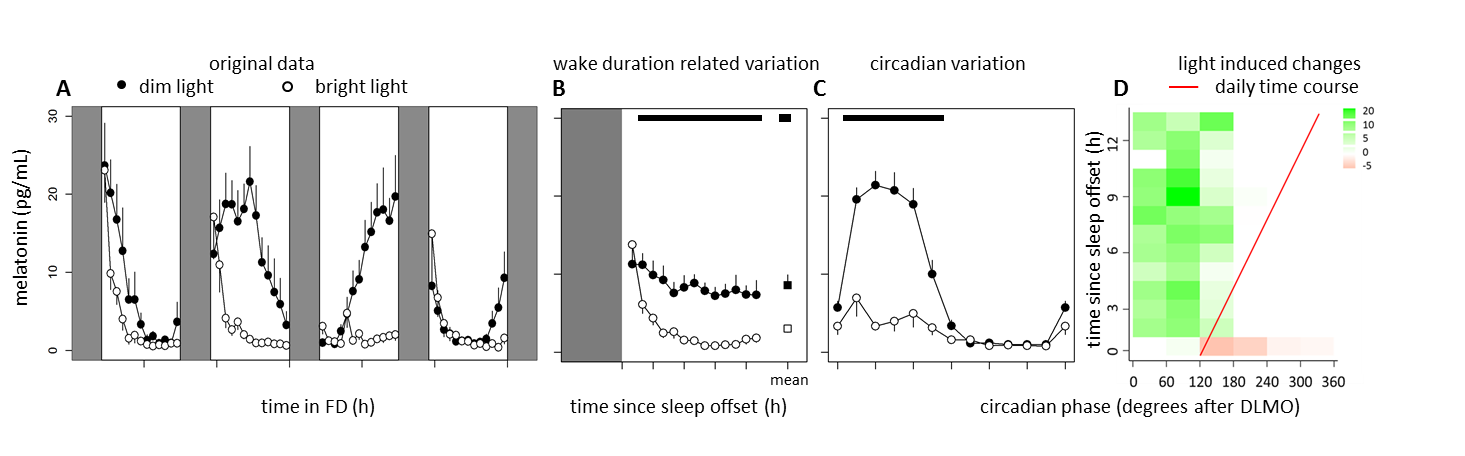
**Figure S3: Data of melatonin concentrations.** Time course of melatonin (A) during the FD protocol. Data replotted as time since sleep offset (B) and circadian phase in degrees after DLMO (C), for melatonin. Contrast analysis describing light induced decrease for all combinations of circadian clock phase and time since sleep offset. Data represent mean ± standard error of the mean, with 7 subjects per group. Black dots indicate data collected in dim light, white dots represent data collected in bright light and black and white squares represent averages over all data points under DL and BL respectively. Red line indicates the expected time course over a regular day. Shaded areas represent scheduled sleep (at 0 lux). Significant differences between light conditions (p<0.05) are indicated by horizontal black bars (B,C) or colored rectangles (D).

It is well established that CBT decreases with increasing melatonin concentrations. Moreover, high intensity light exposure suppresses melatonin production. Melatonin was hourly measured from habitual sleep offset onwards in our design(Lok et al., 2020), therefore a correlation analysis was performed to investigate effects of melatonin on CBT variation (Fig S2). This revealed lowered melatonin concentrations in BL coinciding with decreased CBT levels. A quadratic fit indicated a significant relationship between melatonin and CBT both in dim (p<0.001, R^2^= 0.38) and bright light (p<0.001, R^2^=0.18), with a significantly different constant, suggesting an additional effect of light exposure on CBT (of approximately 0.1 ºC), independent of melatonin suppression (Fig S2). Similar results were found in skin temperature measures at proximal regions, while skin temperature measured in distal regions indicate opposing patterns (Fig S2, Table S2).


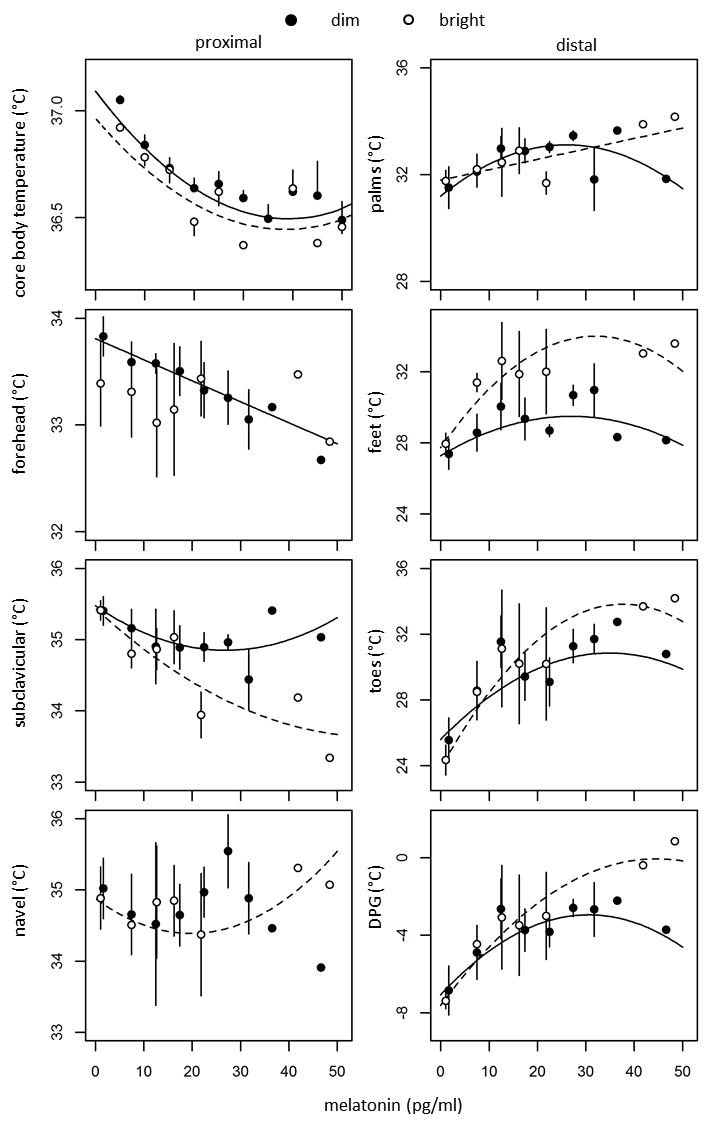


**Figure S4. Melatonin concentrations (pg/ml) plotted against core body temperature, skin temperature measured at the palms, forehead, feet, subclavicular are, toes, navel and DPG (ºC) with data binned in bins of 5 pg/mL (averages ± SEM).** Data represent individual data points of 8 subjects for CBT, and 6 subjects for skin temperature parameters. Black dots indicate data collected in dim light, while white dots represent data collected in BL. High melatonin values in BL are caused by melatonin values immediately after awakening (Lok et al., 2020). A parabolic curve was fitted to predict the relationship between melatonin concentrations and CBT levels, with the solid and dotted line depicting this relationship in dim and bright light respectively.

**Table S2: Summary of model fits on Core Body Temperature, skin temperature measured at the forehead, subclavicular region, navel, palms, feet, toes and DPG.** Both significance and model fits from quadratic or linear fits are indicated by p and R^2^ values. Significant relationships between melatonin concentration and temperature correlates exist for all parameters in both light conditions, except T_forehead_ during bright light, and T_navel_ during dim light exposure.

|  | **dim** | | **bright** | |
| --- | --- | --- | --- | --- |
| **Core Body Temperature** | *p, R^2^* | **<0.001,**  **0.38** | *p, R^2^* | **<0.001,**  **0.18** |
| **Forehead** | *p, R^2^* | **<0.0001,**  **0.22** | *p, R^2^* | >0.05,  0.006 |
| **Clavicles** | *p, R^2^* | **<0.0001,**  **0.15** | *p, R^2^* | **<0.0001,**  **0.24** |
| **Navel** | *p, R^2^* | >0.05,  0.01 | *p, R^2^* | **<0.05,**  **0.06** |
| **Palms** | *p, R^2^* | **<0.0001,**  **0.13** | *p, R^2^* | **<0.05,**  **0.06** |
| **Feet** | *p, R^2^* | **<0.01,**  **0.07** | *p, R^2^* | **<0.0001,**  **0.22** |
| **Toes** | *p, R^2^* | **<0.0001,**  **0.15** | *p, R^2^* | **<0.0001,**  **0.26** |
| **DPG** | *p, R^2^* | **<0.0001,**  **0.19** | *p, R^2^* | **<0.001,**  **0.31** |

CIE (2018) CIE System for Metrology of Optical Radiation for ipRGC-influenced Responses to Light. Cent Bur Vienna, Austria

Lok R, Woelders T, Koningsveld MJ van, Oberman K, Fuhler S, Beersma DGM and Hut RA (2020) Light effects on circadian and homeostatic regulation (i): alertness increases independent of time awake.

Lucas RJ, Peirson SN, Berson DM, Brown TM, Cooper HM, Czeisler CA, Figueiro MG, Gamlin PD, Lockley SW, O’Hagan JB, Price LLA, Provencio I, Skene DJ and Brainard GC (2014) Measuring and using light in the melanopsin age. Trends Neurosci
